# Metal–Organic Frameworks as Room Temperature Chemiresistive Ammonia Gas Sensing Material: A Review

**DOI:** 10.3390/s26113379

**Published:** 2026-05-26

**Authors:** Ehtisham Muhammad, Xiao-Feng Sun, Annum Zia, Ran Sun, Sihai Hu

**Affiliations:** 1 School of Chemistry and Chemical Engineering, Northwestern Polytechnical University, Xi’an 710129, China; ehtisham0039@mail.nwpu.edu.cn (E.M.); husihai@nwpu.edu.cn (S.H.); 2 Shenzhen Research Institute of Northwestern Polytechnical University, Shenzhen 518057, China; 3 National Centre of Bioinformatics, Quaid-e-Azam University, Islamabad 15320, Pakistan

**Keywords:** pristine MOFs, conductive MOFs, MOF-based composites, ammonia (NH_3_) sensing, chemiresistive gas sensors

## Abstract

The growing demand for reliable, real-time detection of ammonia (NH_3_) has accelerated the development of chemiresistive gas sensors, while conventional semiconductors employed as sensing materials in chemiresistive sensors remain constrained by limited selectivity and high operating temperatures (typically 200–400 °C). Among the emerging porous materials, metal–organic frameworks (MOFs) have attracted significant attention as room-temperature NH_3_ sensing materials owing to their structural tunability, enabling precise control over pore chemistry, functionality, and metal centers. However, a comprehensive study specifically focused on MOF-based chemiresistive NH_3_ sensors operating at room temperature remains limited. This review critically targets the investigation of pristine MOFs, conductive MOFs, and MOF-based composites for NH_3_ sensing, with an emphasis on sensing mechanisms, structure–property–performance relationships, stability, selectivity, and environmental effects. Furthermore, rational design strategies and prospects are discussed to provide guidelines for the development of next-generation high-performance room-temperature NH_3_ chemiresistive sensors.

## 1. Introduction

Ammonia (NH_3_) is one of the most widely produced and utilized industrial chemicals globally, playing a central role in agriculture, refrigeration, chemical manufacturing, and emerging energy systems [1]. However, its excessive emission poses significant risks to both environmental sustainability and human health [2,3]. Contemporary assessments indicate that global emissions of reactive nitrogen have escalated to unprecedented levels, with agricultural NH_3_ emissions increasing by approximately 78% over the past four decades, reflecting the intensification of anthropogenic activities and their profound impact on the global nitrogen cycle [4]. Atmospheric NH_3_ contributes to the formation of secondary fine particulate matter (PM_2.5_) through reactions with acidic gases, leading to severe respiratory and cardiovascular impacts, while also causing ecosystem acidification and biodiversity loss [5]. Consequently, there is a growing global demand for highly sensitive, selective, and real-time NH_3_ detection technologies, particularly for applications spanning environmental monitoring, industrial safety, agriculture, and healthcare diagnostics [6,7,8].

Over the last few decades, a broad spectrum of gas-sensing technologies such as electrochemical detectors [9], optical systems [10], surface acoustic wave devices [11,12], calorimetric sensors [13], and chemiresistive gas sensors [14,15] has been investigated to address the growing need for reliable gas monitoring. However, chemiresistive gas sensors operate through a physicochemical transduction mechanism in which interactions between gaseous analytes and the surface of an active sensing material modulate the material’s electronic properties, producing measurable variations in electrical resistance or conductance [15,16]. Chemiresistive gas sensors have emerged as a promising platform due to their simplicity, low cost, compactness, and compatibility with portable devices [17,18,19]. Conventional chemiresistive sensors are primarily based on metal oxide semiconductors (MOSs) [20], conducting polymers [21], carbon-nano tubes [22], and transition metal dichalcogenides (TMDs) [23], which have been extensively investigated for NH_3_ sensing. Among them, MOS-based chemiresistors have been widely employed due to their high advantages, including low costs, simple operating principles, rapid responses, and good reversibility. These materials exhibit excellent sensitivity and fast response characteristics, making them highly suitable for detecting a wide range of gases [24,25,26]. But they require elevated operating temperatures, typically higher than 150 °C, to activate surface reactions [27]. At these temperatures, adsorbed oxygen molecules are ionized into reactive species such as O_2_^−^, O^−^, and O^2−^, which play a crucial role in the sensing mechanism by interacting with target gas molecules [28]. To overcome these limitations, research has increasingly focused on developing sensing materials that operate at room temperatures, enabling lower energy consumption and portable applications, such as carbon-based materials [29] and transition metal dichalcogenides (TMDs) [30]. Carbon-based materials often exhibit limited sensitivity and selectivity due to a lack of available active functional sites, despite their inherently high surface area [31]. Although transition metal dichalcogenides (TMDs) offer high surface area and abundant active sites through exfoliation, they are susceptible to oxidative degradation, which limits their stability [32]. In this regard, metal–organic frameworks (MOFs) have emerged as promising candidates to address the limitations of conventional chemiresistive materials [33].

**Figure 1:**
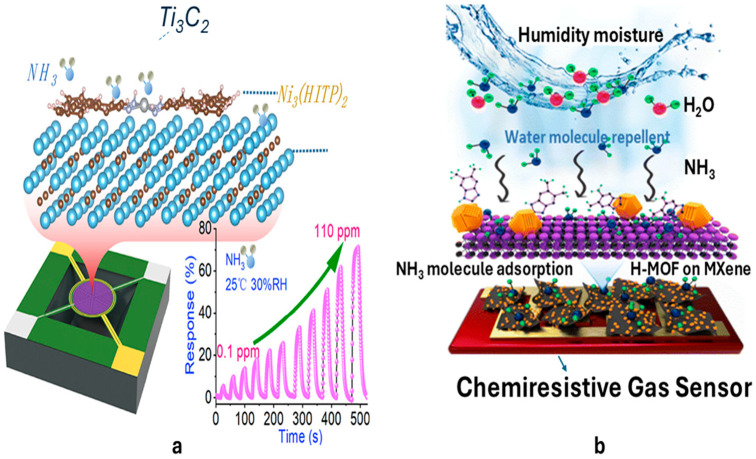
Schematic illustration of chemiresistive NH_3_ sensing using: (**a**) Ni_3_(HITP)_2_/Ti_3_C_2_ composites [50]; (**b**) Hydrophobic ZIF-67/MXene [51]. Copyrights: (**a**) image has been taken with permission from © 2026, American Chemical Society; (**b**) image has been taken and reproduced from © 2024, Royal Society of Chemistry.

In recent years, metal–organic frameworks (MOFs) constitute a new class of crystalline, reticular coordination networks assembled through the linkage of metal ions or metal-oxo clusters with multitopic organic ligands via strong coordination bonds, providing periodically ordered porous architectures at the molecular scale [34,35]. Following the seminal demonstrations of reticular framework construction in 1995 by Yagi et al. [36], metal–organic framework (MOF) chemistry has progressed into a highly developed field encompassing tens of thousands of structurally characterized materials with programmable topology and functionality. The incorporation of MOFs into chemiresistive sensing platforms has significantly broadened their practical applicability across industrial safety monitoring, environmental pollutant detection, and healthcare diagnostics [37,38]. Additionally, the structural diversity of MOFs allows for rational design strategies, including composite formation, conductivity enhancement, and functionalization, which further improve sensing performance [39,40]. MOF-based NH_3_ chemiresistive sensors are generally classified into three main categories: (1) Pristine (non-conductive) MOFs, (2) Conductive MOFs, and (3) MOF-based composites designed to enhance sensing performance at room temperature. In addition to these major classes, several emerging categories have also been explored, including mixed-matrix MOFs [41], bimetallic MOFs [42], and MOF-derived materials [43,44,45]. However, this study primarily focuses on the above three categories due to their dominant contribution to room-temperature NH_3_ chemiresistive sensing performance and the general schematic illustration of sensing using these MOF-based materials can be observed in Figure 1. As owing to their tunable pore structures, high surface areas, and tailorable host–guest interactions, MOF-based resistive sensors enable selective gas adsorption and analyte-induced modulation of electrical conductivity, thereby facilitating sensitive and real-time detection of NH_3_ [46,47,48]. Despite these advantages, the sensing behavior of MOF-based chemiresistive sensors is highly dependent on the intricate interplay between their structural characteristics, physicochemical properties, and charge transport mechanisms [49]. Therefore, a comprehensive understanding of the structure–property–performance relationships is essential to guide the rational design of next-generation NH_3_ sensors.

In this context, this review systematically examines recent progress in room-temperature MOF-based chemiresistive NH_3_ sensors, with an emphasis on sensing mechanisms and critical performance metrics, structural design strategies spanning pristine MOFs, conductive MOFs, and MOF-based composites, as well as the structure–property–performance relationships governing NH_3_ adsorption, transport, charge transfer, and MOF–NH_3_ interactions. In addition, key challenges associated with stability, selectivity, humidity interference, and chemical robustness are critically discussed. Finally, prospects are outlined through rational design strategies, conductive and hybrid MOF architectures, and the development of flexible and wearable room-temperature NH_3_ sensing platforms.

## 2. Mechanism and Critical Merit for Chemiresistive NH_3_ Sensing

Resistive (Chemiresistive) gas sensors operate by monitoring variations in electrical resistance resulting from interactions between gas molecules and the surface of sensing materials [52]. When NH_3_ diffuses into the sensing layer, it adsorbs onto active surface sites and participates in charge transfer processes or reactions with pre-adsorbed oxygen species and these surface interactions induce changes in charge carrier concentration, mobility, and interfacial potential barriers, leading to measurable variations in electrical conductivity [53,54]. The sensing mechanism can be described using a receptor–transducer model, in which the surface chemical reactions act as the reception stage and the resulting electrical resistance change serves as the transduction signal [55]. The generated resistance signals are subsequently processed through external electronic circuits to enable a quantitative determination of gas concentration [56]. The resistance response might increase or decrease, depending on either its n-type or p-type chemiresistive sensor (Table 1).

**Figure 2:**
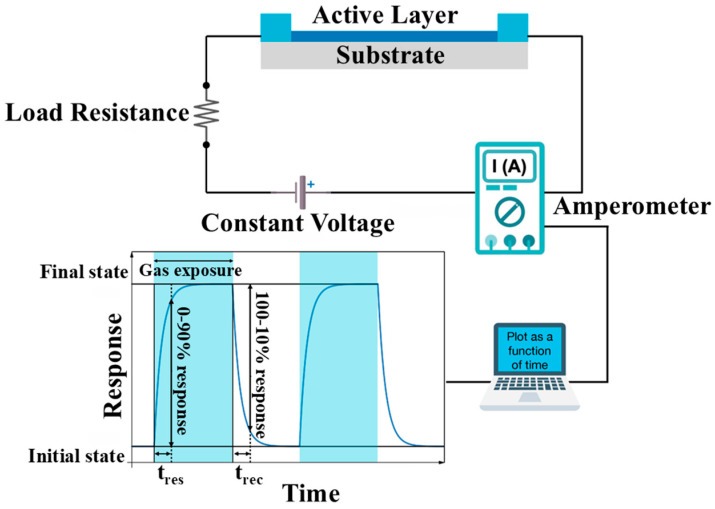
Schematic illustration of a chemiresistive sensing setup, where the active sensing layer is integrated into an electrical circuit with a voltage source and load resistance. The time-dependent electrical response (current, voltage, or resistance) is monitored during gas exposure to record the dynamic sensing behavior [61]. Copyrights: image has been taken and reproduced from © 2024, MDPI.

Although the primarily focus of this study is on chemiresistive NH_3_ sensing, recent advances in image-based and optical MOF sensing platforms, including fluorescent and colorimetric MOF arrays, have demonstrated promising capabilities for visual gas detection and remote monitoring [57]. MOF-based fluorescent sensors exhibit advantages such as high sensitivity, rapid responses, and multidimensional optical signal acquisition due to their tunable porous structures and optical properties [58]. In addition, colorimetric MOF sensors provide simple visual interpretation and spatially resolved sensing through analyte-induced color changes, making them attractive for point of need applications and computer vision-assisted data analysis [59,60]. Therefore, integrating chemiresistive and image-based MOF sensing systems may offer complementary advantages for developing intelligent and real-time NH_3_ monitoring with enhanced visualization, sensitivity, and environmental adaptability. In general, for chemiresistor design (Figure 2), the sensing layer exhibits an initial resistance (R_0_) under ambient conditions, which changes to R_gas_ upon gas exposure due to adsorption-induced modulation of gas concentration and surface band structure. Adsorbed molecules act as electron donors or acceptors [61].

Significant progress has been achieved in the development of MOF-based NH_3_ chemiresistive sensing materials. However, comparatively less attention has been devoted to signal processing, post-processing and data interpretation strategies required for reliable NH_3_ quantification and in practical environments, and chemiresistive responses are often influenced by humidity fluctuations, baseline drift, temperature variation, and electrical noise, which can lead to non-linear sensing behavior and reduced reproducibility [62]. Therefore, appropriate signal conditioning approaches, including baseline correction, filtering, drift compensation, and humidity calibration, are essential for improving sensing accuracy and long-term stability [63,64]. Recently, machine learning-assisted approaches have emerged as promising tools for enhancing gas sensing performance and selectivity. Algorithms such as principal component analysis (PCA), artificial neural networks (ANNs), support vector machines (SVMs), and random forest models have been employed to analyze complex sensor response patterns, suppress noise, compensate for environmental interference, and enable quantitative NH_3_ prediction under mixed-gas conditions [65,66,67]. The integration of advanced signal processing with MOF-based sensing platforms may significantly improve the practical applicability of room-temperature NH_3_ chemiresistive sensors in real-world monitoring systems.

**Table 1:** Classification of chemiresistive sensors’ reactions to different gases [68].

Response Behavior	p-Type	n-Type
Reducing Gas (H_2_, H_2_S, NH_3_, CO, CH_4_)	Increase in resistance	Decrease in resistance
Oxidizing Gas (SO_2_, NO_2_, NO, O_3_)	Decrease in resistance	Increase in resistance
Dominant charge carrier	Holes	Electrons

The performance of chemiresistive ammonia (NH_3_) gas sensors is evaluated using key parameters, including sensitivity, selectivity (or cross-sensitivity), reversibility, limit of detection (LOD), response and recovery times, and stability [69]. (1) Sensitivity refers to the relative change in sensor output upon exposure to NH_3_. (2) Selectivity describes the ability to distinguish a specific analyte in the presence of target gas NH_3_. (3) Reversibility indicates the capability of the sensor to return to its original state after NH_3_ removal. (4) The LOD represents the minimum detectable NH_3_ gas concentration above the baseline signal. (5) Response time is defined as the duration required to reach 90% of the final signal after exposure, while (6) recovery time corresponds to the time needed to return to 90% of the initial baseline once the NH_3_ gas is removed. (7) Stability reflects the reproducibility of sensor performance over repeated cycles, whereas long-term stability or lifetime denotes the ability to maintain consistent performance over extended periods [69,70,71]. These sensors can even detect trace gas concentrations (sub-ppm levels) with rapid response times, often within tens of seconds, making them highly suitable for real-time NH_3_ monitoring [50].

The post-processing strategies were implemented (especially baseline correction, filtering, and humidity and drift compensation) to improve the signal-to-noise ratio (SNR), particularly under low-concentration and high-humidity conditions where low-frequency noise strongly affects signal stability [72]. In gas sensors, such noise is a key factor limiting detection reliability, yet it is often less addressed than material optimization. Recent signal restoration concepts from other fields, such as the Inverted Haze Density Correction Prior (IHDCP) used in image dehazing, demonstrate how adaptive correction of degraded signals can improve reconstruction quality [73]. Similar denoising-inspired strategies may offer useful perspectives for improving the robustness of chemiresistive sensing signals.

## 3. Structural Design of MOFs for NH_3_ Sensing

### 3.1. Pristine (Non-Conductive) MOFs

Pristine metal–organic frameworks (MOFs) represent the earliest and most fundamental class of sensing materials employed in chemiresistive ammonia (NH_3_) detection. These materials are typically electrically insulating or semiconducting in nature, and their sensing response originates primarily from adsorption-induced modulation of charge transport rather than intrinsic conductivity [1,74,75]. Due to their high surface area, tunable pore structure, and strong gas interactions, these MOFs provide abundant adsorption sites for NH_3_ molecules, enabling detectable resistance variations even in the absence of intrinsic electrical conductivity. However, their insulating framework significantly limits charge carrier transport, resulting in a relatively low response and often requiring elevated operating temperatures or hybridization with conductive additives for practical chemiresistive performance [76].

**Figure 3:**
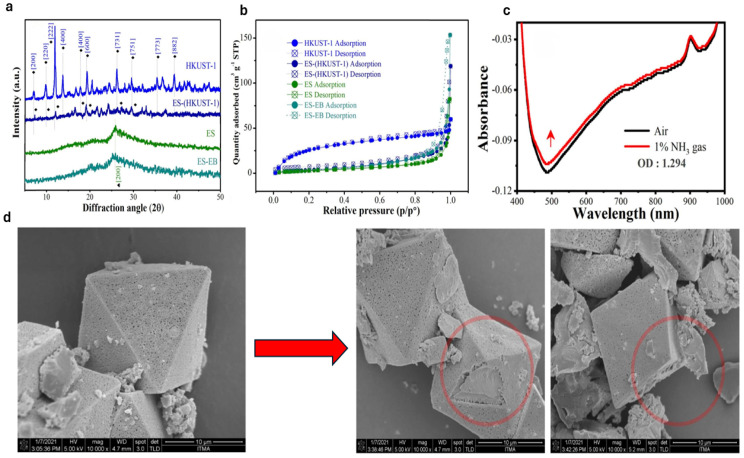
(**a**) XRD patterns, (**b**) N_2_ adsorption–desorption isotherms, (**c**) absorbance shift upon 1% NH_3_ exposure, and (**d**) FESEM images of HKUST-1 before (**left**) and after (**right**) NH_3_ exposure (at 10,000X) [77]. Copyrights: images have been taken with permission from © 2022, Elsevier.

Although the gas sensing performance of non-conductive MOFs are lower than conductive ones and very few studies have been done in this domain, a study [77] presented the influence of HKUST-1 on NH_3_ sensing at room temperatures. Figure 3a presents the XRD pattern of the corresponding sample, where the presence of intense and sharp diffraction peaks, particularly at the (222) plane, confirms the high crystallinity of HKUST-1. Figure 3b shows the N_2_ adsorption–desorption isotherms of the respective samples, where HKUST-1 exhibits a steep uptake at low relative pressure, confirming its predominant microporous structure. The absorbance shift of HKUST-1 upon exposure to 1% NH_3_ gas illustrated in Figure 3c indicates a pronounced interaction between NH_3_ molecules and the framework. The FESEM images of HKUST-1 in Figure 3d reveal noticeable surface defects on the octahedral crystals after NH_3_ adsorption (highlighted in red), while the overall morphology remains largely intact even at high NH_3_ concentrations, indicating structural stability of the framework.

The intrinsically insulating nature of such MOFs severely limits charge transport, resulting in weak signal transduction, reduced sensitivity, and often compromised room-temperature performance [78]. These inherent limitations restrict their standalone applicability in high-performance sensing platforms. Consequently, to overcome these challenges and enhance the electrical response, recent research has progressively shifted toward the development of conductive MOF systems, where improved charge mobility enables more efficient and amplified transduction of NH_3_ adsorption events into measurable electrical signals [79].

### 3.2. Conductive MOFs

Conductive MOFs (C-MOFs) are a highly promising class of materials for room-temperature NH_3_ sensing due to their intrinsic electrical conductivity combined with structural tunability and porosity [80]. Unlike conventional insulating MOFs, conductive MOFs possess extended π-conjugation and metal–ligand electronic coupling, which enable the efficient charge transport pathways necessary for high-performance chemiresistive sensing applications [81]. The conductive MOF-based sensors outperform their pristine counterparts in terms of signal-to-noise ratio and operational stability [82]. The most high-performance NH_3_ sensors are dominated by 2D layered and 1D chain-like conductive MOFs [83]. One-dimensional (1D) C-MOFs are chain-structured coordination polymers that enable directional charge transport, making them ideal candidate for room-temperature chemiresistive gas sensing [84]. A study [33] reported the synthesis of three distinct 1D conductive Cu-MOFs (Cu-MOF-1, Cu-MOF-2, and Cu-MOF-3) with different topological architectures via a solvothermal route using varied solvent systems. The corresponding chemiresistive sensors, fabricated by a drop-coating method, exhibited excellent NH_3_ sensing performance at room temperature, showing a pronounced response toward 100 ppm NH_3_ under room-temperature conditions.

**Figure 4:**
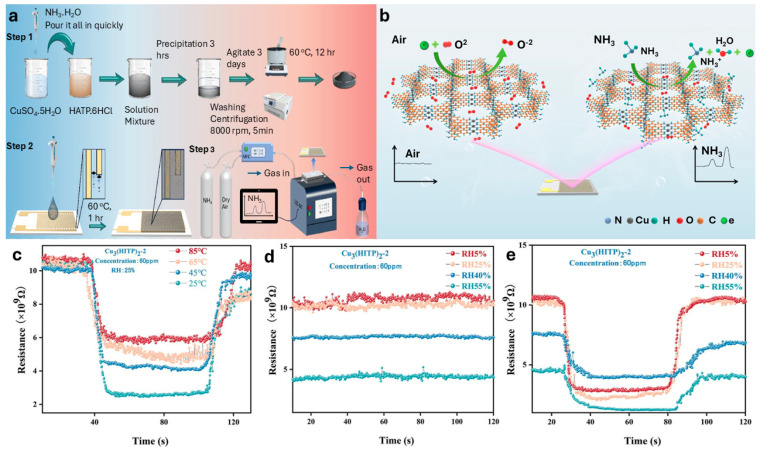
(**a**) Step.1: Synthesis of Cu_3_(HITP)_2_; Step.2: Sensor fabrication; and Step.3: Gas-sensing performance evaluation. (**b**) Illustration of the gas-sensing mechanism of the Cu_3_(HITP)_2_-based sensor. (**c**) Response curves toward 60 ppm NH_3_ at various operating temperatures under 25% relative humidity (RH) conditions. (**d**) Effect of RH on the baseline resistance of the sensor at 25 °C. (**e**) Response curves toward 60 ppm NH_3_ at different RH levels at 25 °C [85]. Copyrights: images have been taken and reproduced from © 2025, Elsevier.

Conductive MOF architectures represent a critical design strategy for advancing next-generation room-temperature NH_3_ sensing technologies. The study in [85] presented an application of 2D π-conjugated Cu MOF. A chemiresistive sensor fabricated by drop-casting two-dimensional layer-stacked Cu_3_(HITP)_2_ nanomaterials onto interdigitated gold electrodes, forming a conductive sensing film. The device exhibited a high response of 91.4% toward 100 ppm NH_3_ at 25 °C, with a rapid response and recovery times of 26 s and 20 s, respectively, and a low detection limit of ~15 ppb, demonstrating efficient room-temperature NH_3_ sensing performance. In Figure 4a, step.1 illustrates the synthesis route of the material. Step.2 illustrates the fabrication process of the NH_3_ gas sensor while in step.3, the real-time sensor resistance was continuously monitored using a gas-sensing analysis system (CGS-MT). The gas-sensing mechanism is primarily governed by surface charge transfer interactions, as illustrated in Figure 4b. The response curves of the Cu_3_(HITP)_2_-based sensor toward 60 ppm NH_3_ at different operating temperatures can be observed in Figure 4c. The effect of humidity on sensor performance, reflected by variations in baseline resistance at relative humidity levels of 5%, 25%, 40%, and 55%, is presented in Figure 4d. The response curves of the sensor toward 60 ppm NH_3_ at varying humidity levels are presented in Figure 4e; the sensor exhibits responses of 73.7% and 77.8% at relative humidity levels of 5% and 25%, respectively.

Apart from 2D π-conjugated conductive MOFs, the extended aromatic ligand-based MOFs also exhibits good performance for NH_3_ sensing. The Cu–OHTBN exhibits high crystallinity with a surface area of 307 m^2^ g^−1^, a narrow bandgap of 0.064 eV, and room-temperature conductivity of 2.35 × 10^−3^ S cm^−1^. In NH_3_ sensing, it shows a linear response (2–80 ppm), a low detection limit of 0.061 ppm, and a fast response time of 0.52 min (80 ppm, room temperature), demonstrating efficient semiconductive chemiresistive performance [48]. These C-MOFs significantly enhanced chemiresistive gas sensing performance as compared to the pristine (non-conductive) MOFs being observed. Furthermore, their performance can be further improved through a composite-based design and hybridization with conductive substrates, leading to superior sensitivity, lower detection limits, and faster response–recovery dynamics [86]. Overall, C-MOFs represent a critical advancement in MOF-based NH_3_ sensing and provide a strong foundation for next-generation sensor development.

### 3.3. MOF-Based Composites for Enhanced NH_3_ Sensing

The integration of MOFs with conductive phases, such as metal oxides, carbon nanostructures (e.g., graphene, CNTs), or conducting polymers (like polyaniline (PANI) and polypyrrole (PPy)), synergistically combines the high surface area and selective adsorption capability of MOFs with higher charge transport from the secondary component [87,88]. This heterostructure design facilitates enhanced adsorption–desorption kinetics, improved charge transfer at the interface, and amplified sensor response toward NH_3_ [89]. Moreover, interfacial interactions, including p–n junction formation, Schottky barriers, and hydrogen bonding, play a crucial role in modulating sensing performance, particularly in terms of sensitivity and selectivity [90,91]. Apart from other composites, the ZIF-67-based composites have demonstrated superior performance in chemiresistive NH_3_ sensing and are widely recognized within the category of composite-based MOFs due to their synergistic integration with conductive materials [92]. The intrinsic properties of ZIF-67, including a high surface area, rich Co^2+^ active sites, and a strong affinity toward electron-donating NH_3_ molecules, enable effective adsorption; however, its limited electrical conductivity restricts its direct sensing performance [93]. To address this, hybridization with conductive matrices such as reduced graphene oxide (rGO) or conducting polymers significantly enhances charge transport and facilitates efficient surface charge transfer interactions. The study in [94] reported a ZIF-67/rGO composite synthesized via a facile hydrothermal method, where the integration of the porous MOF with a highly conductive graphene network facilitated efficient charge transfer and enhanced NH_3_ adsorption, resulting in a response of 1.22 ± 0.02 (20 ppm) and 4.77 ± 0.15 (50 ppm), with response/recovery times of 46.5 ± 2.12 s and 66.5 ± 2.12 s, and a low detection limit of 74 ppb, demonstrating stable, reproducible, and humidity-tolerant sensing performance. Another study [95] reported a ZIF-8@CuO@PEDOT:PSS nanostructured composite, where dual heterojunctions formed between ZIF-8, CuO, and the conductive polymer network enable enhanced NH_3_ adsorption and rapid charge transfer. The sensor exhibits an exceptionally high response of 9954 toward 50 ppm NH_3_, with fast response/recovery times of 8/2 s, along with excellent stability and selectivity under ambient conditions, attributed to the synergistic interaction of the porous MOF architecture, catalytic metal oxide sites, and the π-conjugated polymer matrix.

**Figure 5:**
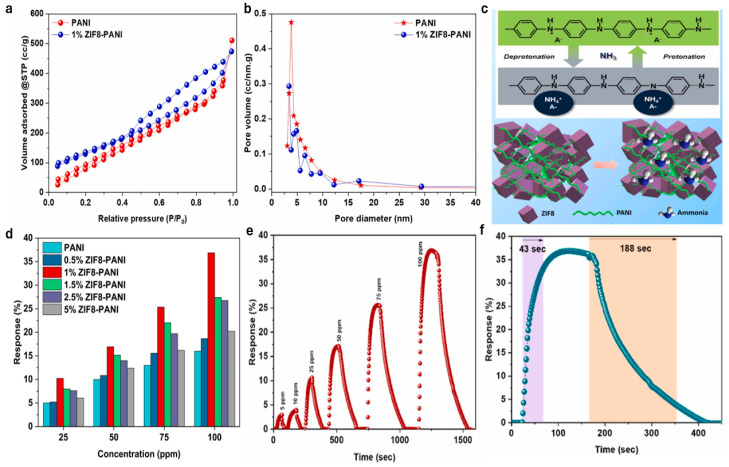
(**a**) Nitrogen (N_2_) adsorption–desorption isotherms. (**b**) Pore size distribution profiles of polyaniline and the 1 wt% ZIF-8/PANI composite. (**c**) Schematic illustration of the sensing mechanism of polyaniline and the ZIF-8/PANI composite under air and NH_3_ environments. (**d**) Comparative NH_3_ sensing performance of ZIF-8/polyaniline composites with 0.5–5 wt% loading. (**e**) Response curves of the 1 wt% ZIF-8/polyaniline-based NH_3_ sensor. (**f**) Response and recovery time profile of the 1 wt% ZIF-8/polyaniline sensor toward 100 ppm NH_3_ [96]. Copyrights: images have been taken with permission from © 2025, Elsevier.

The ZIF-67–polymer composites (e.g., with polyaniline) leverage acid–base interactions between NH_3_ and the polymer backbone, further strengthening the sensing response. The study in [96] reported the fabrication of a room-temperature NH_3_ chemiresistive sensor based on polyaniline and its nanocomposite with ZIF-8, synthesized via a facile in situ chemical polymerization approach. The optimized sensor, based on 1 wt% ZIF-8 loading, exhibits a sensing response ranging from 0.6 to 36.8% toward 5–100 ppm NH_3_, with a detection limit of 5 ppm and a rapid response time of 18 s, demonstrating stable and sensitive room-temperature ammonia detection. In Figure 5a, BET analysis shows that polyaniline exhibits a surface area of 477.034 m^2^ g^−1^, which increases to 488.086 m^2^ g^−1^ upon incorporation of 1 wt% ZIF-8, indicating enhanced available surface sites. The BJH analysis in Figure 5b, shows that polyaniline has a pore volume of 0.683909 cc g^−1^ and a pore radius of 1.91693 nm, while the 1 wt% ZIF-8/PANI composite exhibits a pore volume of 0.694603 cc g^−1^ and a pore radius of 1.7067 nm, as the incorporation of ZIF-8 increases pore volume and decreases the average pore size due to structural modification and partial pore blocking within polyaniline. Figure 5c illustrates the sensing mechanism upon NH_3_ exposure, i.e., protonated polyaniline (emeraldine salt) undergoes deprotonation via interaction with NH_3_ at N^+^–H sites, forming NH_4_^+^ and converting it into the insulating emeraldine base form. Figure 5d compares the NH_3_ sensing response of pure polyaniline and ZIF-8/PANI nanocomposites with varying ZIF-8 loadings across different ammonia concentrations, demonstrating their concentration-dependent sensing performance. The 1 wt% ZIF-8/polyaniline sensor exhibits response values of 2.9%, 3.9%, 10.5%, 17.08%, 25.57%, and 36.86% toward 5, 10, 25, 50, 75, and 100 ppm NH_3_, respectively, as shown in Figure 5e. The sensor exhibits response and recovery times of 43 s and 188 s, respectively, as shown in Figure 5f, which are shorter than those of pure polyaniline, indicating improved sensing dynamics.

Overall, MOF-based composites have demonstrated a substantial advancement in room-temperature NH_3_ chemiresistive sensing by overcoming the intrinsic limitations of pristine MOFs, particularly their limited conductivity and slow charge transport. Additionally, MOF-based composites outperform simple composite MOFs because they introduce synergistic multi-phase interfaces that simultaneously enhance conductivity, adsorption kinetics, and charge transport, leading to higher sensitivity, faster response–recovery behavior, and improved stability in NH_3_ detection [97]. The sensing characteristics of these categories are strongly influenced by operating conditions such as humidity, temperature, and NH_3_ concentration range. The comparative analysis can be observed in Table 2. Future studies should emphasize standardized testing protocols under identical humidity, temperature, and NH_3_ concentration conditions to enable more reliable quantitative comparison among different MOF-based sensing platforms.

## 4. Structure–Property–Performance Relationships in NH_3_ Sensing

### 4.1. Influence of Porosity and Structural Architecture on NH_3_ Adsorption and Transport

Establishing clear structure–property relationships between MOF structural features and NH_3_ adsorption is essential for rational adsorbent design. MOF–NH_3_ surface interactions are typically probed using in situ techniques such as neutron powder diffraction (NPD) for adsorption site identification, infrared (IR) spectroscopy for binding interactions, and solid-state NMR (ssNMR) for local chemical environments, complemented by INS, EPR, and UV–Vis spectroscopy for dynamic and electronic insights [104]. Moreover, the pore size and porosity of MOFs are key parameters controlling NH_3_ adsorption and diffusion. Microporous MOFs (<2 nm) enhance NH_3_ uptake due to a high surface area and strong adsorption potential; however, they often suffer from pore diffusion limitations and increased mass transfer resistance, which can slow adsorption–desorption kinetics and degrade response/recovery times [105,106]. In contrast, a mesoporous and hierarchical MOF offers reduced internal diffusion barriers, promotes faster intraparticle mass transport (including Knudsen and surface diffusion), and thus improves adsorption kinetics, leading to superior dynamic responses for real-time NH_3_ sensing applications [107,108]. The comparative assessment of sensing performance for representative room-temperature MOF-based NH_3_ chemiresistive sensors reported in the literature is summarized in Table 2.

**Figure 6:**
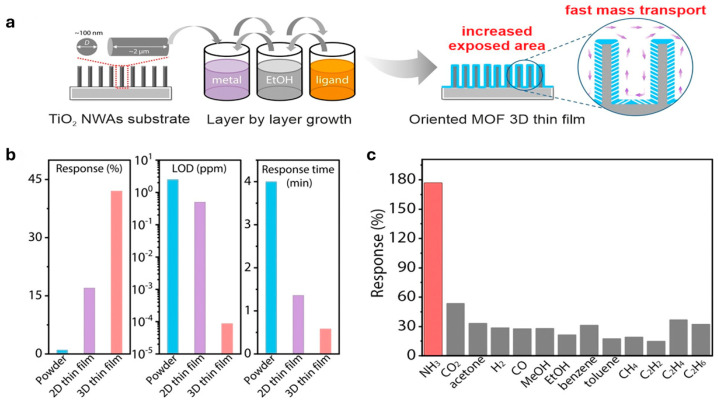
(**a**) Schematic illustration of the synthetic route for growing hierarchical 3D Cu-HHTP films on preordered TiO_2_ nanowire array substrates. (**b**) Comparison of room-temperature NH_3_ sensing performance of Cu-HHTP in powder, 2D film, and 3D film configurations. (**c**) Selectivity profile of the Cu-HHTP 3D film sensor toward NH_3_ relative to interfering gases at 100 ppm [109]. Copyrights: images have been taken with permission from © 2024, American Chemical Society.

The structural architecture plays a critical role in governing NH_3_ transport and sensing performance. For instance, the study in [109] presented TiO_2_ nanowire array-supported 3D Cu-HHTP films that exhibited responses 161 and 2.5 times higher than pristine Cu-HHTP powder and 2D films, respectively, toward 1 ppm NH_3_, while reducing the response time to 35 s at 100 ppm compared to 240 s and 81.6 s for powder and 2D films. This enhancement is attributed to the 3D hierarchical architecture and Cu-HHTP/TiO_2_ interfacial coupling, which increase accessible active sites and promote more efficient mass and charge transfer (Figure 6a–c). More broadly, such architecture-dependent effects extend to interpenetrating MOF topologies (IMOFs), where multiple independent networks (1D, 2D, or 3D) coexist within the same structure without covalent bonding, leading to complex architectures and tunable properties. Topological analysis typically treats each net separately, though multiple valid representations may exist due to structural complexity [110,111]. The surface chemistry of MOFs plays a decisive role in NH_3_ adsorption, where open metal sites, defect sites, and polar functionalities enhance uptake through Lewis acid–base interactions and hydrogen bonding. In bimetallic Cu_1_Co_x_BTC systems (e.g., Cu_1_Co_1_BTC), Co incorporation effectively tunes these surface properties, generating synergistic active sites that improve adsorption capacity, sensitivity, and stability [112].

### 4.2. Influence of Charge Transfer and MOF-NH_3_ Interactions on Sensing Response

**Figure 7:**
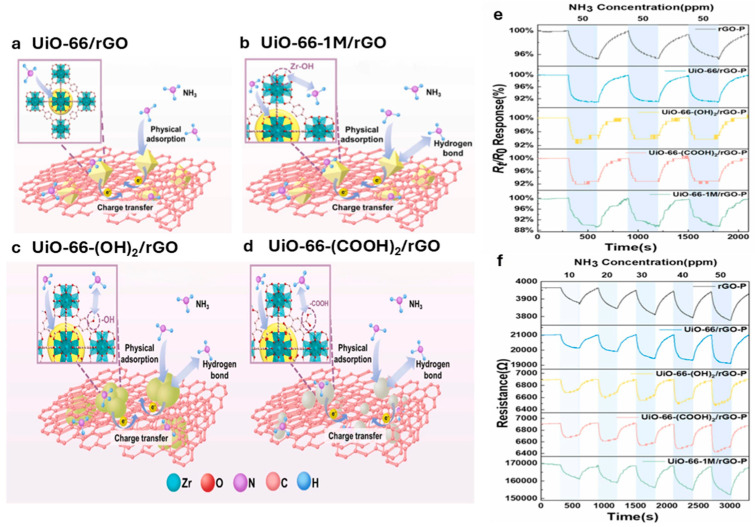
Schematic illustration of the NH_3_ sensing mechanisms in (**a**) pristine UiO-66, (**b**) acid-modified UiO-66–1M, (**c**) hydroxyl-functionalized UiO-66-(OH)_2_, and (**d**) carboxyl-functionalized UiO-66-(COOH)_2_. Comparison of concentration-dependent sensing behavior: (**e**) the cyclic response profiles of rGO-P and MOF/rGO-P sensors toward 50 ppm NH_3_, and (**f**) the dynamic resistance response curves of rGO-P and MOF/rGO-P sensors over 10–50 ppm NH_3_ [117]. Copyrights: images have been taken with permission from © 2025, Elsevier.

The sensing mechanism in MOF-based chemiresistive sensors is fundamentally governed by the interfacial charge transfer modulation triggered upon NH_3_ chemisorption. The comparative assessment of sensing performance for representative room-temperature MOF-based NH_3_ chemiresistive sensors reported in the literature is also summarized in Table 3. To interpret these performance trends, it is important to consider the underlying transduction mechanism. In conductive MOFs and their heterostructure composites, the adsorption of NH_3_ acting as a reducing analyte, induces electron injection into the framework, thereby shifting the charge carrier concentration and the Fermi level [49,113,114]. In p-type semiconducting architectures, this electron donation facilitates hole–electron recombination, increasing electrical resistance, whereas in n-type systems, it enhances conductivity; this electronic transduction allows for high-precision monitoring of NH_3_ at sub-ppm concentrations [115,116]. Consistent with this mechanism, a study [117] presented MOF/rGO hybrid sensors, which exhibited enhanced room-temperature NH_3_ sensing performance relative to pristine rGO, delivering higher response and faster response kinetics, while plasma treatment further reduced the response time through improved MOF–rGO interfacial coupling and surface activation that promote NH_3_ adsorption. Functional group engineering significantly modulates NH_3_ adsorption and sensing selectivity in UiO-66/rGO hybrids. In Figure 7a, NH_3_ adsorption in pristine UiO-66/rGO occurs through pore-confined physisorption and hydrogen bonding with residual oxygen-containing groups on rGO. The acid treatment enhances the adsorption affinity by increasing accessible active sites, as shown in Figure 7b. In Figure 7c, hydroxyl-functionalized UiO-66-(OH)_2_/rGO strengthens NH_3_ capture through hydrogen bonding while rGO facilitates rapid electron transport, accelerating adsorption equilibrium and improving response kinetics. Figure 7d further demonstrates that carboxyl-functionalized UiO-66-(COOH)_2_/rGO enhances NH_3_ uptake through polarized carboxyl sites and stronger donor–acceptor interactions, collectively highlighting the critical role of functional groups in governing adsorption strength and selectivity. As shown in Figure 7e, both rGO-P and MOF/rGO-P sensors exhibited nearly unchanged responses over three consecutive sensing cycles, demonstrating good repeatability and indicating that an oxygen plasma treatment did not compromise sensor stability. Furthermore, Figure 7f shows that the sensors maintained distinct response signals toward 10 ppm NH_3_, confirming preserved sensitivity and a reliable low-concentration detection capability. This stable and reproducible sensing behavior is attributed to sustained interfacial charge transfer and efficient electron transport within the MOF/rGO heterostructure, which underpin consistent signal transduction upon repeated NH_3_ adsorption–desorption cycles. Overall, these findings demonstrate that the synergistic host–guest interactions at MOF interfaces improve charge separation and electron mobility, enabling fast response/recovery and enhanced sensitivity. Therefore, performance enhancement is not only governed by adsorption strength but also by efficient interfacial charge transfer pathways [118,119].

## 5. Stability, Selectivity, and Environmental Effects

The practical deployment of MOF-based chemiresistive NH_3_ sensors critically depends on their stability, selectivity, and resilience under realistic environmental conditions. Although MOFs offer high surface area and tunable adsorption properties, their performance can be significantly influenced by moisture, temperature, and long-term operational factors, which must be carefully addressed for reliable sensing applications [126].

### 5.1. Moisture and Humidity Interference in NH_3_ Sensing

Humidity is one of the most challenging interfering factors in NH_3_ detection, as water molecules competitively adsorb on active sites and alter the sensing response. In many MOFs, especially hydrophilic frameworks such as HKUST-1, water molecules coordinate with metal centers or occupy pore channels, thereby reducing the availability of adsorption sites for NH_3_ and degrading sensor sensitivity. Additionally, the formation of hydrogen-bonding networks between H_2_O and NH_3_ can alter adsorption thermodynamics and lead to signal instability [77,127].

To mitigate these effects, different strategies have been employed. (1) Pore size engineering: The engineering pore apertures and channel dimensions in MOFs that moderate sizes (typically ~4–7 Å) have been shown to promote selective NH_3_ adsorption by optimizing confinement effects and diffusion kinetics, while mitigating competitive H_2_O adsorption under humid environments [128,129]. (2) Defect engineering: The introduction of structural defects, particularly missing linker induced open metal sites, significantly enhances NH_3_ adsorption by increasing the density of coordinatively unsaturated Lewis acid sites and facilitating an enhanced binding affinity toward NH_3_, thereby improving chemiresistive sensing performance [130,131]. (3) Surface functionalization with hydrophobic groups: The incorporation of hydrophobic functional groups (e.g., –CH_3_, –CF_3_, and silane-based moieties) into MOF frameworks via linker modification or post-synthetic treatment reduces competitive H_2_O adsorption by weakening framework–water interactions, while maintaining NH_3_ adsorption sites and facilitating humidity-tolerant sensing performance [51]. (4) Core–shell protective structures: The construction of core–shell architectures, where MOFs are encapsulated within hydrophobic shells or conductive polymer coatings (e.g., polyaniline), has been demonstrated to effectively suppress humidity interference by acting as a protective barrier layer, thereby preserving active sites while maintaining stable sensing performance under humid conditions [132,133].

### 5.2. Thermal and Chemical Stability

The stability of MOFs is primarily governed by the strength of metal–ligand coordination bonds, where enhanced bond strength between metal nodes and organic linkers significantly improves resistance to hydrolysis and structural degradation [134]. In particular, zirconium-based frameworks such as UiO-66 exhibit exceptional chemical robustness due to strong Zr–O coordination arising from hard acid–base interactions, making them highly stable under aqueous and harsh operating conditions [135]. In contrast, MOFs constructed from relatively weak metal–ligand coordination bonds such as Zn- or Co-based ZIFs and certain p-block metal frameworks (e.g., In^3+^-based MOFs like MFM-300(In)) are prone to structural degradation upon NH_3_ exposure, where strong NH_3_ coordination induces ligand displacement and framework collapse, leading to a loss of crystallinity and sensing performance [136,137].

Chemical stability is often influenced by exposure to basic gases such as NH_3_, which can induce framework degradation through competitive coordination and ligand displacement. For instance, MOFs containing divalent metal nodes (e.g., Zn^2+^- or Cu^2+^-based frameworks such as MOF-5 and HKUST-1) exhibit limited stability toward NH_3_, where strong NH_3_ coordination to metal centers disrupts metal–ligand bonds, leading to a loss of porosity [138]. This instability arises because NH_3_, as a strong Lewis base, can displace coordinated linkers or attack labile metal–ligand bonds, ultimately resulting in framework distortion and degradation [139].

### 5.3. Long-Term Stability and Degradation Mechanisms

The MOF-based NH_3_ chemiresistive sensors have demonstrated sensing performance under controlled laboratory conditions; most reported studies primarily emphasize short-term repeatability, whereas systematic long-term stability assessments and field-validation studies remain comparatively limited. Moisture exposure and prolonged environmental operation can induce structural degradation in MOFs through mechanisms such as metal-node hydrolysis [140], linker displacement [141], framework collapse, and pore blockage, particularly under humid conditions [142]. In study [143], an IC-MOF/biofabric sensor demonstrated excellent room-temperature NH_3_ sensing performance, including a high response of 14.7 toward 1 ppm NH_3_, a low detection limit of 36 ppb, and remarkable selectivity coefficients exceeding 5.12 against common interferents, though prolonged exposure to humid operating environments may still induce hydrolysis of metal–ligand coordination bonds and gradual framework degradation. Such moisture-induced instability can progressively deteriorate active adsorption sites and electrical pathways, thereby affecting long-term sensing reproducibility and operational durability despite the improved environmental adaptability of the sensor platform. Accordingly, the adoption of standardized accelerated aging protocols under controlled humidity, temperature, and cyclic gas exposure conditions is recommended to avoid misleading short-term performance interpretation and to ensure an accurate evaluation of long-term operational stability [144,145].

## 6. Perspectives and Design Guidelines

The rapid evolution of MOF-based NH_3_ sensors has demonstrated remarkable progress in sensitivity, selectivity, and room-temperature operation; however, translating these advances into practical and scalable technologies requires rational material design, device integration, and system-level optimization.

### 6.1. Rational Design Strategies for High-Performance NH_3_ Sensors

Future MOF design must move beyond trial-and-error synthesis toward structure-guided engineering, where adsorption affinity, conductivity, and diffusion pathways are simultaneously optimized. For instance, recent work on conductive MOFs such as Cu_3_(HITP)_2_ demonstrates that intrinsic conductivity combined with ordered π–d conjugation enables ppb-level detection (~0.015 ppm) with a fast response at room temperature, highlighting the importance of electronic band structure tuning in sensor design [85]. Similarly, advanced Hoffmann-type MOFs exhibit an ultra-high NH_3_ adsorption capacity and detection limits down to ~25 ppb with an ~5 s response time, indicating that precise control of coordination environment and binding energetics is critical for achieving high sensitivity [146]. Therefore, future strategies should focus deeply on: (a) tuning metal ligand chemistry for optimal NH_3_ binding affinity, (b) designing hierarchical porosity for balanced adsorption diffusion kinetics, and (c) engineering electronic pathways for efficient charge transport.

### 6.2. Integration of Conductive MOFs and Hybrid Architectures

A major trend is the integration of conductive MOFs with hybrid systems to overcome intrinsic limitations. While pristine MOFs offer a high adsorption capacity, their poor conductivity restricts their sensing performance [97]. For instance, ZIF-8/CNT composites exhibit stable NH_3_ sensing at room temperature with strong humidity tolerance (45–70% RH) due to the synergistic effect of adsorption (ZIF-8) and charge transport (CNTs) [147]. Similarly, a Cu_3_(HHTP)_2_/rGO nanocomposite demonstrated a dual-gas sensing capability with stable NH_3_ detection over a wide concentration range (10–50,000 ppm), along with excellent flexibility, long-term stability (~40 days), and tolerance to high humidity (~75% RH), confirming the robustness of conductive MOF carbon hybrid systems [148]. Future design guidelines should therefore emphasize: (a) band alignment optimization for efficient charge transfer, and (b) multi-functional architectures combining adsorption, catalysis, and conductivity.

### 6.3. Flexible, Wearable, and Room-Temperature Sensing Devices

The next generation of NH_3_ sensors is shifting toward flexible, low-power, and wearable platforms for real-time environmental and biomedical monitoring. Recent advances demonstrate the feasibility of integrating MOFs into flexible substrates and wireless sensing systems. A notable example is a wireless wearable MOF-based sensor patch that operates without batteries and enables continuous gas monitoring, highlighting the transition toward portable and user-friendly sensing technologies [149]. Key future directions include: (a) the development of flexible MOF thin films and coatings, (b) integration with wearable electronics and IoT systems, and (c) achieving stable room-temperature operation without external heating.

## 7. Conclusions

This review article presented the development of MOF-based NH_3_ sensors which has clearly indicated that sensing performance is governed by the synergistic interplay between structural design, surface chemistry, and charge transport mechanisms. It highlighted and evaluated the sensing performance for NH_3_ sensing using pristine MOFs, conductive MOFs, and MOF-based composites at room temperature. Key insights from recent studies demonstrate that pore engineering and topology optimization directly control NH_3_ diffusion and adsorption kinetics, while open metal sites and functional groups enhance binding affinity via Lewis acid–base interactions. Despite these advances, challenges related to humidity interference and long-term stability remain significant barriers to real-world deployment. These limitations can be mitigated via “design guidelines”, through defect engineering, hydrophobic functionalization, and hybrid composite design, which enhance environmental tolerance and maintain stable charge transport pathways. Moreover, the integration of MOFs into flexible and wearable devices, along with their compatibility with low-power sensing platforms, underscores their potential for next-generation applications. MOFs offer a uniquely tunable platform for NH_3_ sensing, combining a high surface area, adjustable pore structures, and versatile chemical functionality. With continued progress in rational material design, hybrid integration, and scalable device engineering, MOF-based sensors are poised to play a pivotal role in advanced environmental monitoring, industrial safety, and smart sensing technologies.

## Figures and Tables

**Table 2:** Comparative analysis of pristine MOFs, conductive MOFs, and MOF-based composites for NH_3_ sensing at room temperature (RT).

Category	Advantages	Limitations	Operating Conditions	Ref.
Pristine MOFs	High porosity, abundant active sites	Poor conductivity	Mostly RT, moderate humidity	[98,99]
Conductive MOFs	Fast electron transport, improved response	Complex synthesis, stability issues	RT to mild heating	[100,101]
MOF-based Composites	Synergistic sensing enhancement, higher sensitivity	Interfacial complexity	Broad NH_3_ range, better humidity tolerance	[102,103]

**Table 3:** Comparative performance analysis of various MOF-based NH_3_ chemiresistive sensors.

Materials	Sensitivity (ppm^−1^)	Response Time (s)	Recovery Time (min)	Ref.
**Pristine MOF** ZIF-8	0.133 (10 ppm)	63	0.75	[120]
**C-MOFs** CuBHT	0.15 (100 ppm)	58	~119	[121]
Cu_3_(HHTP)_2_ nanorod film	0.154 (3 ppm)	3	~30	[122]
**MOF-Based Composites**				
TiO_2_@Cu-HHTP	1.62 (100 ppm)	35	15	[123]
PAIN/UiO-66	25.4 (100 ppm)	25	0.23	[124]
Cu-BTC/Carbon-graphene	0.0538 (100 ppm)	~4.60	~45	[125]
ZIF-67/rGO	~4.77 (50 ppm)	46.5	~1.10	[94]
H-MOF6/MXene	1.337 (10 ppm)	25	3.2	[51]

## Data Availability

Not applicable.
